# FgSsn3 kinase, a component of the mediator complex, is important for sexual reproduction and pathogenesis in *Fusarium graminearum*

**DOI:** 10.1038/srep22333

**Published:** 2016-03-02

**Authors:** Shulin Cao, Shijie Zhang, Chaofeng Hao, Huiquan Liu, Jin-Rong Xu, Qiaojun Jin

**Affiliations:** 1 State Key Laboratory of Crop Stress Biology for Arid Areas, College of Plant Protection, Northwest A&F University, Yangling, Shaanxi, China; 2 Dept. of Botany and Plant Pathology, Purdue University, West Lafayette, Indiana, USA

## Abstract

*Fusarium graminearum* is an important pathogen of wheat and barley. In addition to severe yield losses, infested grains are often contaminated with harmful mycotoxins. In this study, we characterized the functions of *FgSSN3* kinase gene in different developmental and infection processes and gene regulation in *F. graminearum*. The *FgSSN3* deletion mutant had a nutrient-dependent growth defects and abnormal conidium morphology. It was significantly reduced in DON production, *TRI* gene expression, and virulence. Deletion of *FgSSN3* also resulted in up-regulation of *HTF1* and *PCS1* expression in juvenile cultures, and repression of *TRI* genes in DON-producing cultures. In addition, *Fgssn3* was female sterile and defective in hypopodium formation and infectious growth. RNA-seq analysis showed that FgSsn3 is involved in the transcriptional regulation of a wide variety genes acting as either a repressor or activator. FgSsn3 physically interacted with C-type cyclin Cid1 and the *cid1* mutant had similar phenotypes with *Fgssn3*, indicating that FgSsn3 and Cid1 form the CDK-cyclin pair as a component of the mediator complex in *F. graminearum*. Taken together, our results indicate that *FgSSN3* is important for secondary metabolism, sexual reproduction, and plant infection, as a subunit of mediator complex contributing to transcriptional regulation of diverse genes.

Wheat head blight (FHB) disease is one of the most destructive diseases of wheat. *Fusarium graminearum* is a major causal agent of FHB in the world^1^ and it also infects other small grain crops, including barley and maize^1^,^2^. In addition to yield losses, this pathogen is a producer of deoxynivalenol (DON), zearalenone, and other mycotoxins. DON is a potent inhibitor of eukaryotic protein synthesis and zearalenone is an estrogenic mycotoxin. Both of them are harmful to human and animals.

*F. graminearum* initiates plant infection when ascospores land on flowering wheat heads. The fungus could form hyphopodia for direct penetration of plant tissues. DON is a phytotoxin and it is, in fact, the first virulence factor identified in *F. graminearum*^3^,^4^. The *TRI5* trichodiene synthase gene is expressed as early as in hyphopodia^5^. Other important pathogenicity factors that have been characterized in *F. graminearum* include genes involved in various signal transduction pathways, metabolism, and developmental processes^6^,^7^,^8^,^9^,^10^,^11^,^12^,^13^,^14^,^15^,^16^. Interestingly, a number of them, such as the protein kinase genes related to cAMP signaling and three mitogen-activated protein (MAP) kinase pathways also are involved in the regulation of DON biosynthesis and sexual reproduction^10^,^14^,^15^. Other protein kinase genes that are important for DON production, plant infection, and sexual reproduction include *SNF1*^17^*, FGK3*^18^, and several genes characterized in the kinome study of *F. graminearum*^19^. One of them is FGSG_04484 that encodes a protein orthologous to the cyclin-dependent protein kinase (CDK) Ssn3 (=Srb10 or Ume5) in yeast and CDK8 in human.

Orthologs of Ssn3 are conserved from yeast to humans and involved in regulation of RNA polymerase II (Pol II)-dependent gene transcription^20^,^21^. Ssn3 is the only protein kinase in the yeast mediator complex, which functions as a bridge between gene-specific transcription regulators and Pol II machinery at the promoter region^22^. Like Cdk8 in humans, kinase activity of Ssn3 is related to binding with C-type cyclin Ssn8 (=Srb11). In yeast, Ssn3 is a regulator of global transcription and affects many important cellular processes, such as filamentous growth and cell cycle progression^23^,^24^,^25^. In humans, Cdk8 has been shown to repress the transcription of immune response genes^26^, and activate genes within the serum response network^27^.

In *S. cerevisiae*, *SSN3* was originally identified as a suppressor of the C-terminal domain (CTD) truncation of Pol II^28^,^29^. Together with its cyclin Ssn8, yeast Ssn3 forms a stable complex with Srb8 and Srb9, which is one sub-module of the mediator complexes^30^. As a nonessential subunit of the mediator complex, Ssn3 regulates gene transcription probably by phosphorylation of the CTD of Pol II^20^. Deletion of *SSN3* decreases the stability of meiotic mRNAs and induces the expression of genes repressed by glucose and mating type-specific genes. *SSN3* also is involved in the regulation of genes related to stress responses and nutrient utilization^23^,^31^.

Although *SSN3* orthologs are well conserved in plant pathogenic ascomycetes, none of them has been functional characterized. This study aims to determine the function of *FgSSN3* in plant infection and other developmental processes in *F. graminearum*. Deletion of *FgSSN3* resulted in medium-dependent growth defects, loss of female fertility, reduced hyphopodium formation, and defects in infectious growth. In DON-producing cultures, the *Fgssn3* mutant was repressed in *TRI* gene expression but increased in the transcription of genes related to aurofusarin biosynthesis. RNA-seq analysis also showed that FgSsn3 negatively or positively regulated the transcription of different subsets of genes. FgSsn3 physically interacted with C-type cyclin Cid1 and likely functions as the CDK-cyclin pair in the mediator complex to regulate the expression of various genes important for growth, differentiation, and pathogenesis in *F. graminearum*.

## Results

### The *Fgssn3* mutant has nutrient-dependent growth defects

**Figure 1:**
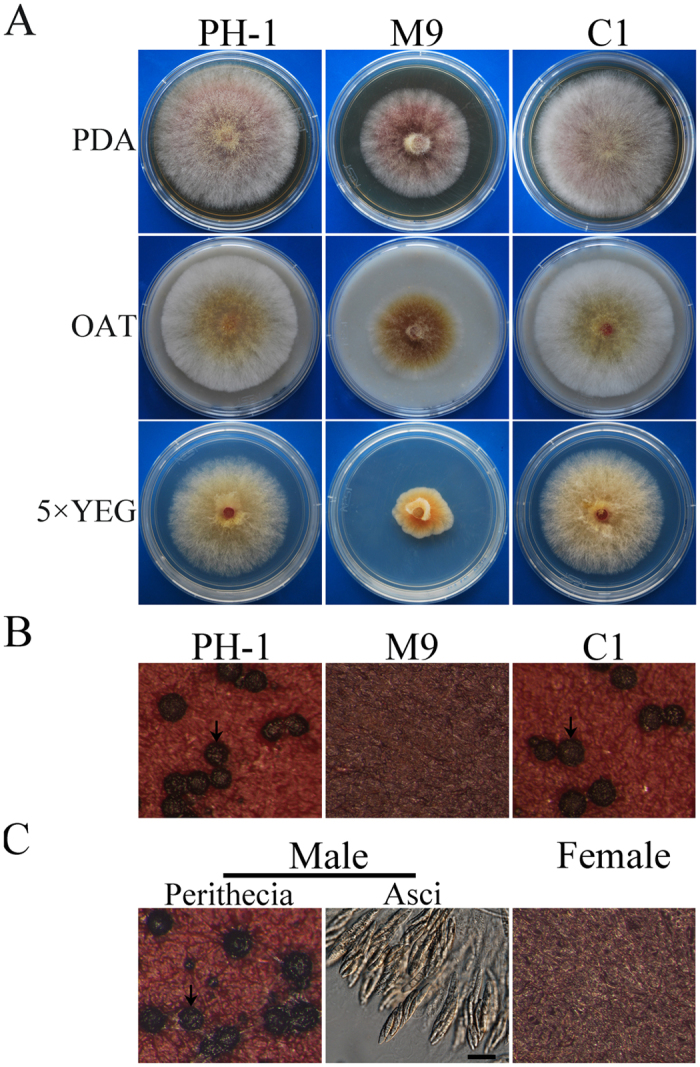
Defects of the *Fgssn3* mutant in growth and sexual reproduction. (**A**) Colonies of wild-type (PH-1), *Fgssn3* deletion mutant (M9), complemented strain (C1) cultured on PDA, OTA and 5 × YEG medium for 3 days. (**B**) Self-crossing plates of PH-1, M9, and C1 at 14 days post-fertilization. Arrows point to perithecia. (**C**) Mating cultures of the *Fgssn3* mutant used as the male (left) or female (right) crossed with the *mat1-1-1* mutant were examined for perithecia and ascospore formation 2 weeks post- fertilization. Bar = 20 μm.

The *SSN3* ortholog in *F. graminearum* FGSG_04484.3 named as *FgSSN3* in this study encodes a 453 amino acid protein. Sequence alignment revealed that *SSN3* orthologs are well conserved in filamentous fungi. The *Fgssn3* mutant was generated with the split-marker approach in a previous study of the *F. graminearum* kinome^19^. In this study, three putative *Fgssn3* mutants, M5, M7, and M9 were further confirmed by Southern blot analysis (Fig. S1). All the *Fgssn3* mutants had the same phenotype although only data for M9 were described below. Compared with the wild type, the *Fgssn3* mutant was reduced in growth rate and produced fewer and shorter aerial hyphae (Fig. 1A). Interestingly, the growth defect of the *Fgssn3* mutants was nutrient-dependent. In comparison with the growth rate of PH-1, the *Fgssn3* mutants had the most significant reduction (56%) on 5 × YEG and less reduction (13%) on oatmeal agar (OTA) and PDA (27%) (Table 1). Whereas 5 × YEG is a synthetic medium, OTA and PDA are medium with natural substrates.

### *FgSSN3* is essential for female fertility

When assayed for sexual reproduction on carrot agar medium, the wild type formed small black perithecia 7 days post-perithecial induction, and produced cirrhi after 10 days. However, no perithecia were observed in *Fgssn3* mutant (Fig. 1B), indicating the importance of *FgSSN3* during sexual reproduction. To determine whether its mating defects were related to male or female fertility, the *Fgssn3* mutant was out-crossed with the *mat1-1-1* deletion mutant^9^. When *Fgssn3* was used as the male, fertile perithecia with normal ascospores were produced (Fig. 1C). In contrast, no perithecia were formed when *Fgssn3* was used as the female (Fig. 1C), indicating that *FgSSN3* is essential for female fertility but dispensable for male fertility.

### The *Fgssn3* mutant is de-repressed in conidiophore development

**Figure 2:**
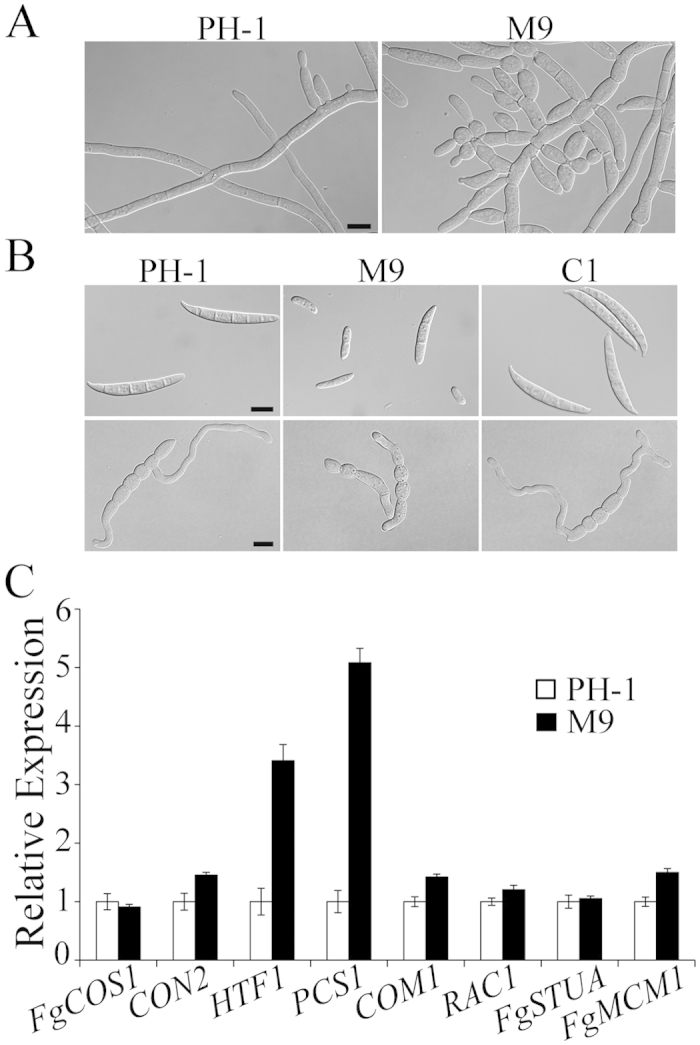
Defects of the *Fgssn3* mutant in conidiogenesis and conidium morphology. (**A**) CMC cultures of the wild type (PH-1) and *Fgssn3* mutant (M9) after incubation for 12 h. Bar = 10 μm. (**B**) Conidia of PH-1, M9, and the complemented transformant C1 were examined for difference in morphology (upper row) and germ tube growth after incubation in YEPD for 6 h (lower row). Bar = 10 μm. (**C**) Expression levels of genes related to conidiation were assayed by qRT-PCR assays. RNA samples were isolated from 12 h YEPD cultures of PH-1 and M9. For each gene, its expression level in PH-1 was arbitrarily set to 1. Mean and standard deviation were calculated with data from three biological replicates.

Although the *Fgssn3* mutant was reduced in growth rate, it produced the same amount of conidia as the wild type (Table 1). Microscopic examination revealed that the *Fgssn3* mutant tended to produce phialides and conidia earlier than the wild type. After 12 h incubation in CMC medium, clusters of phialides were observed in *Fgssn3* but not in the wild type (Fig. 2A). However, mutant conidia were shorter and had fewer septa than the wild type (Fig. 2B). Approximately one-third of the mutant conidia also lacked typical tip or foot cells. Nevertheless, *Fgssn3* conidia could germinate normally although germ tube growth was reduced (Fig. 2B), which is consistent with its reduced growth rate.

We transformed the full-length *FgSSN3* allele into the *Fgssn3* mutant. The resulting *Fgssn3*/*FgSSN3* transformant had the wild-type growth rate (Fig. 1A) and normal conidium morphology (Fig. 2B). The defects of the mutant in sexual reproduction and plant infection also were complemented by the ectopic integration of *FgSSN3*, indicating that deletion of *FgSSN3* was directly responsible for defects of *Fgssn3* mutants.

### *HTF1* and *PCS1* are up-regulated in the *Fgssn3* mutant

In *F. graminearum*, a number of genes, including *FgMCM1*, *FgSTUA, PCS1, HTF1, COM1, FgCOS1, RAC1*, and *CON2* are known to be important for conidiation^32^,^33^,^34^,^35^,^36^,^37^,^38^,^39^. To assay their expression in the *Fgssn3* mutant, RNA samples were isolated from 12 h CMC cultures. Compared with PH-1, transcript abundance of other genes was not affected, but the expression levels of *HTF1* and *PCS1* were up-regulated over 3- and 5-fold, respectively, in the *Fgssn3* mutant (Fig. 2C). The *PCS1* transcription factor gene plays a role in regulating proper production of conidia. Overexpression of *PCS1* increased the formation of intercalary phialides^34^. *HTF1* encodes a conserved homeobox transcription factor important for conidiogenesis and phialide formation^35^. The up-regulation of these two genes in *Fgssn3* may be responsible for the de-repression of phialide formation and conidiation in 12 h CMC cultures.

### *FgSSN3* is important for plant colonization and infectious growth

**Figure 3:**
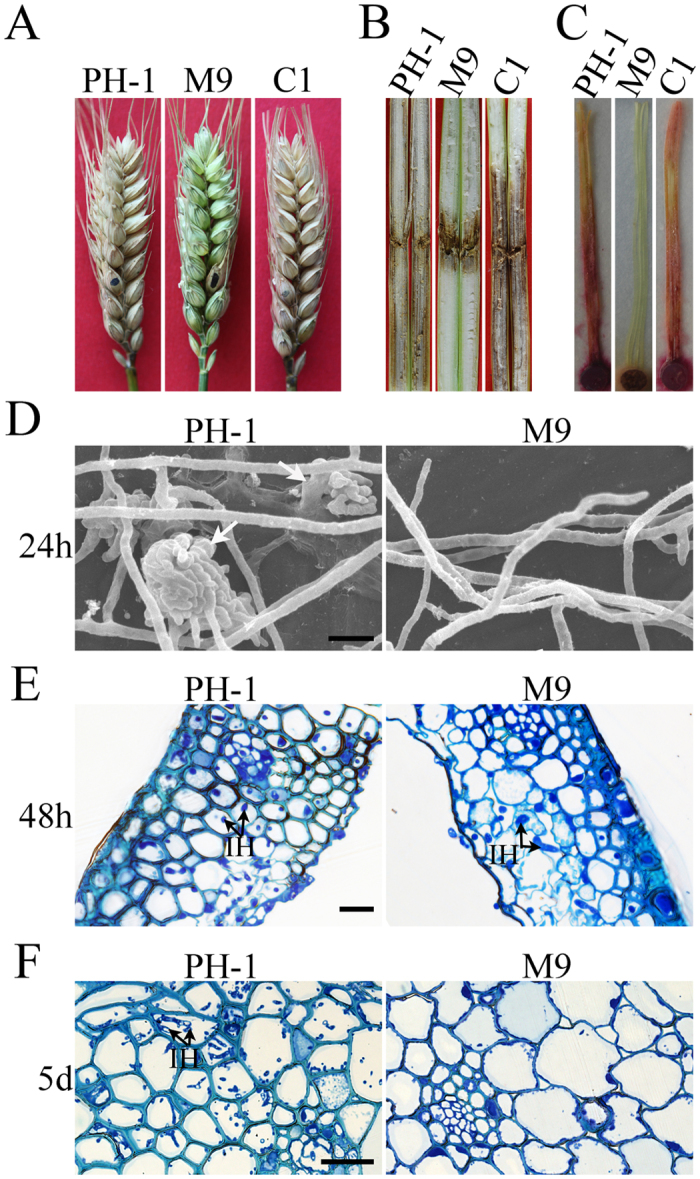
Defects of the *Fgssn3* mutant in plant infection. (**A**) Flowering wheat heads were drop-inoculated with conidia from the wild type (PH-1), *Fgssn3* mutant (M9), and complemented strain (C1). Black dots mark the inoculated spikelets. Photographs were taken 14 days post-inoculation (dpi). (**B**) Corn stalks were inoculated with toothpicks dipped in conidia of the same set of strains and examined for stalk rot symptoms 14 dpi. (**C**) Corn silks were inoculated with blocks of cultures of the same set of strains. Photographs were taken 5 dpi. (**D**) Lemma from the spikelets inoculated with PH-1 and M9 were examined by SEM 24 hpi. Hyphopodia formed on the inner surface are marked with white arrows. Bar = 10 μm. (**E**) Infectious hyphae (IH) formed by PH-1 and M9 inside lemma tissues 48 hpi. Bar = 50 μm. (**F**) Thick sections of rachis tissues directly below the inoculated spikelet were examined for infectious growth 5 dpi. In samples inoculated with PH-1, abundant hyphal growth was observed. No infectious hyphae (IH) were observed in the rachis inoculated with M9. Bar = 50 μm.

In infection assays with flowering wheat heads, the *Fgssn3* mutant developed typical disease symptoms only on the inoculated kernels but never spread to neighboring spikelets at 14 days post-inoculation (dpi) (Fig. 3A). The average disease index of the *Fgssn3* mutant M9 and PH-1 was 0.7 and 11.3, respectively (Table 1), which was approximately a 90% reduction in virulence. Therefore, *FgSSN3* is essential for disease spreading and colonization of wheat head tissues. Similar results were obtained in infection assays with corn stalks (Fig. 3B) and silks (Fig. 3C). Stalk rot and discoloration were restricted to the inoculated sites. These results suggest that *FgSSN3* may play an important role in infectious growth in plant tissues.

To further determine the function of *FgSSN3* in plant infection, we examined the infection processes by scanning electron microscopy (SEM) and light microscopy. At 24 hours post infection (hpi), the wild-type strain formed penetration structures on wheat glumes (Fig. 3D). Under the same conditions, *Fgssn3* was significantly reduced in hyphopodium formation. In fact, hyphopodia were rarely observed in samples inoculated with the mutant (Fig. 3D). Nevertheless, infectious hyphae were observed in lemma tissues inoculated with both the wild type and *Fgssn3* mutant at 48 hpi although the extent of invasive growth was significantly reduced in the latter (Fig. 3E). By 5 dpi, fungal growth was not observed in the rachis below or above the spikelets inoculated with the *Fgssn3* mutant (Fig. 3F). Under the same conditions, abundant intracellular hyphae were observed in the vascular and other tissues of the rachis in samples inoculated with PH-1 (Fig. 3F). These results further indicate that *FgSSN3* is important for infectious growth and spreading from the inoculated spikelet to the rachis and nearby spikelets, which is consistent with its disease index being less than 1.

### *FgSSN3* positively regulates DON biosynthesis during plant infection and in DON-inducing culture conditions

Because of its importance as a virulence factor, we assayed DON production in the *Fgssn3* mutant. In wheat kernels with scab symptoms collected 14 dpi, over 1000 ppm DON was detected in samples inoculated with PH-1 (Table 1). In contrast, DON concentration was less than 50 ppm in samples inoculated with the *Fgssn3* mutants (Table 1). To confirm this observation, we assayed DON production in rice grain cultures as described^40^. DON production was barely detectable in rice grains inoculated with *Fgssn3* (Table 1).

**Figure 4:**
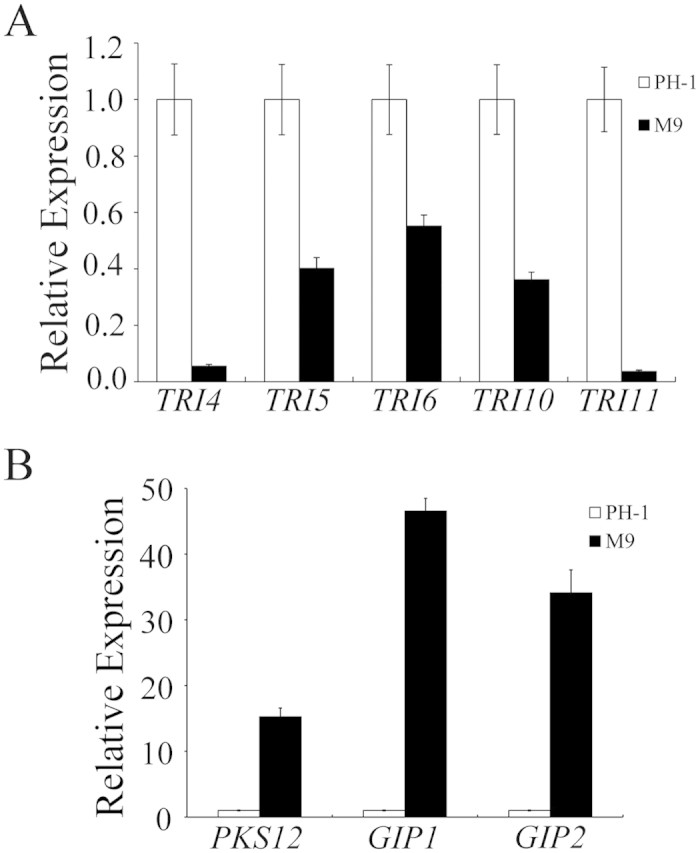
Assays for expression levels of selected genes related to trichothecene and aurofusarin biosynthesis by qRT-PCR. The expression level of each gene in PH-1 was arbitrarily set to 1. Mean and standard deviation were calculated with data from three biological replicates. (**A**) Expression of *TRI4, TRI5, TRI6, TRI10*, and *TRI11* in the wild-type strain PH-1 and *Fgssn3* mutant M9. RNA samples were isolated from DON-producing cultures (containing 5 mM arginine). (**B**) Expression of *PKS12, GIP1*, and *GIP2* in PH-1 and M9. RNA samples were isolated from DON-producing cultures containing 5 mM arginine.

We also assayed the expression levels of the *TRI4*, *TRI5*, *TRI6*, *TRI10*, and *TRI11* genes in DON-inducing cultures containing 5 μM arginine by qRT-PCR assay. In comparison with that of the wild type, the expression level of *TRI4*, *TRI5*, *TRI6*, *TRI10*, and *TRI11* was reduced in the *Fgssn3* mutant for approximately 20, 2.5, 2, 3, and 30 folds, respectively (Fig. 4A). These results showed that *FgSSN3* plays an important role in the regulation of DON biosynthesis under DON-inducing conditions.

### Aurofusarin biosynthesis is negatively regulated by *FgSSN3*

Because the *Fgssn3* mutant appeared to have enhanced reddish pigmentation than PH-1 on PDA and OTA cultures (Fig. 1A), we assayed the expression levels of three genes related to aurofusarin synthesis, the polyketide synthase genes *PKS12* and two putative laccase genes *GIP1* and *GIP2*^41^, with RNA samples used for assaying *TRI* gene expression. In comparison with the wild type, the expression levels of these three genes were increased in the *Fgssn3* mutant (Fig. 4B). The expression of *GIP1* and *GIP2* was upregulated over 30-folds (Fig. 4B), suggesting that *FgSSN3* negatively regulates aurofusarin biosynthesis.

### Expression and localization of FgSsn3-GFP fusion

**Figure 5:**
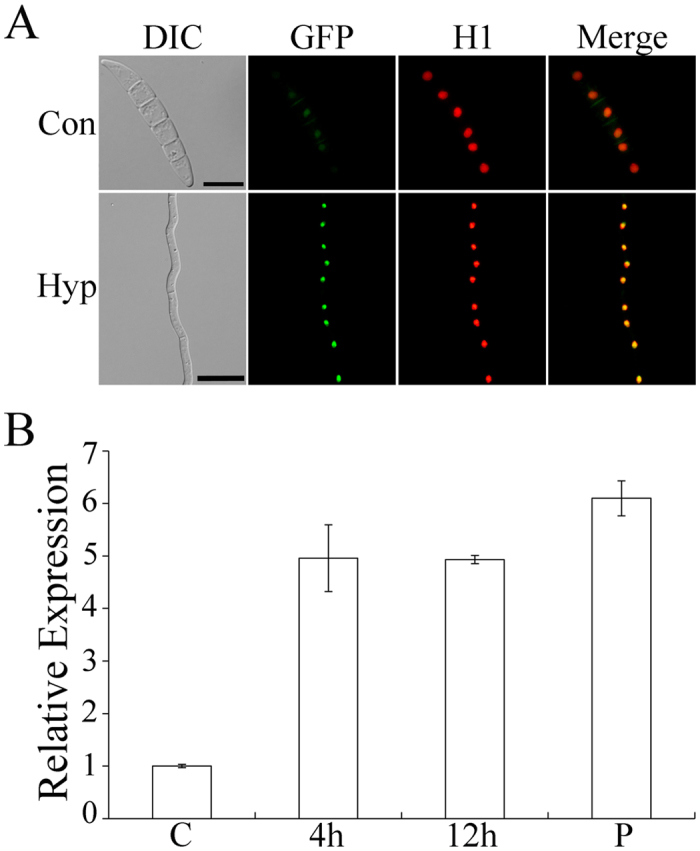
Localization and expression of *FgSSN3*. (**A**) Conidia and hyphae of the *Fgssn3*/*FgSSN3*-GFP H1-mCherry transformant were examined by DIC and epifluorescence microscopy. Bar = 20 μm. (**B**) Relative expression level of *FgSSN3* in conidia (**C**), 4 or 12 h germlings, and perithecia (P). Mean and standard deviation were calculated with data from three replicates.

To determine the expression and localization of FgSsn3, we generated an *FgSSN3*-EGFP fusion construct under the control of its native promotor and transformed it into the *Fgssn3* mutant. In the resulting transformant, although the mutant phenotypes were complemented, no or only faint GFP signals were observed in the nucleus. We then cloned the *FgSSN3*-EGFP fusion construct behind the strong constitutive promotor RP27 that is derived from the *Magnaporthe grisea* ribosomal protein 27^42^,^43^, and transformed it into the *Fgssn3* mutant. In the resulting transformants, GFP signals were observed in the nucleus in conidia and hyphae (Fig. 5A).

We also assayed *FgSSN3* expression in PH-1 by qRT-PCR with RNA samples isolated from conidia, 4 h, or 12 h germlings, and mature perithecia. In comparison with conidia, the expression level of *FgSSN3* was increased approximately 5-fold in 4 h or 12 h germlings and mature perithecia (Fig. 5B), indicating that *FgSSN3* may be constitutively expressed in different growth and developmental stages except in conidia.

### Kinase activity is essential for the function but not subcellular localization of FgSsn3

**Figure 6:**
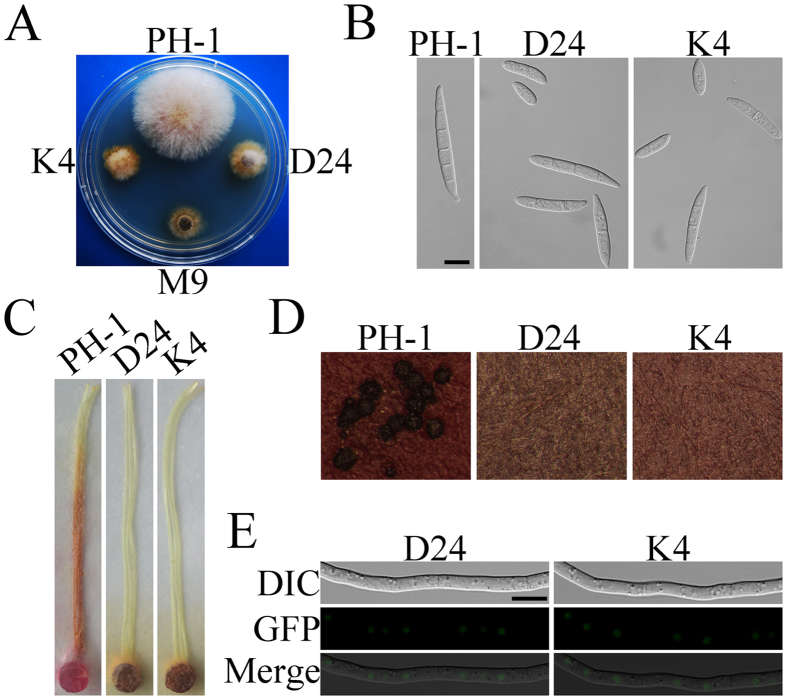
Functions and Localization of FgSsn3^D191A^–GFP and FgSsn3^K71R^-GFP. (**A**) Two-day-old 5 × YEG cultures of the wide type (PH-1), *Fgssn3* mutant (M9), *Fgssn3/FgSSN3*^D191A^*-GFP* transformant (D24), and *Fgssn3/FgSSN3*^K71R^-GFP transformant (K4). (**B**) Conidia of PH-1, D24, and K4 in 4-day-old CMC cultures. Bar = 10 μm. (**C**) Corn silks inoculated with PH-1, D24, and K4 were examined 5 dpi. (**D**) Mating cultures of PH-1, D24 and K4 were examined 2 weeks post-induction for sexual reproduction. (**E**) Hyphae of D24 and K4 were examined by DIC and epifluorescence microscopy. Bar = 10 μm.

To determine whether the kinase activity is essential for FgSsn3 function and localization, we generated the *FgSSN3*^D191A^-GFP and *FgSSN3*^K71R^-GFP alleles and transformed them into the *Fgssn3* mutant. The D191 and K71 residues of FgSsn3 are equivalent to D290 and K183 of *S. cerevisiae* Srb10, respectively, which are highly conserved amino acids in the kinase domain and essential for its kinase activity^44^,^45^. The *Fgssn3/FgSSN3*^D191A^-GFP transformants D8 and D24, and the *Fgssn3/FgSSN3*^K71R^-GFP transformants K4 and K8 had similar defects with the original *Fgssn3* mutant in growth rate (Fig. 6A), conidium morphology (Fig. 6B), plant infection (Fig. 6C), and sexual reproduction (Fig. 6D). However, both FgSsn3^D191A^–GFP and FgSsn3^K71R^-GFP fusion proteins still localized to the nucleus (Fig. 6E). These results suggested that the kinase activity is essential for its function but dispensable for its subcellular localization.

### FgSsn3 is a component of the mediator complex in *F. graminearum*

**Figure 7:**
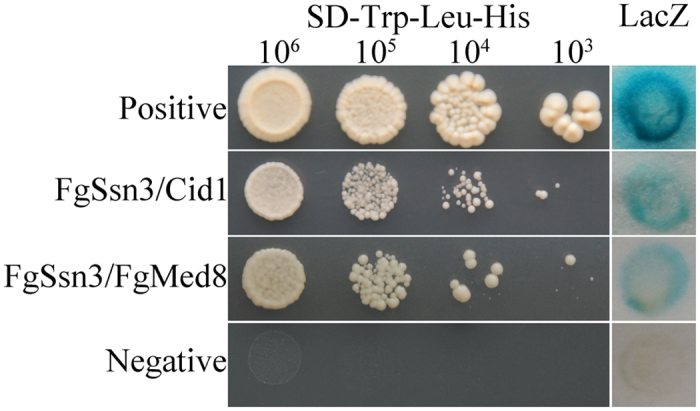
Yeast two-hybrid assays for the interaction between FgSsn3 and Cid1 or FgMed8. Different concentrations (cells/ml) of the yeast transformants expressing the *FgSSN3* prey and Cid1 or FgMed8 bait constructs were assayed for growth on SD-Leu-Trp-His plates and β-galactosidase (LacZ) activities. Positive and negative controls were provided in the BD Matchmaker library construct kit.

In the budding yeast, Ssn3 interacts with Ssn8 (a C-type cyclin), Srb8 (Med12), and Srb9 (Med13) to form the kinase module of the mediator complex^46^. Orthologs of Ssn8, Srb8, and Srb9 and many other components of the yeast mediator complex are conserved in *F. graminearum*. The ortholog of yeast *SSN8*, *CID1*, has been characterized in an earlier study^47^. To confirm that FgSsn3 is also as a component of the mediator complex in *F. graminearum*, we constructed the Cid1 bait and FgSsn3 prey constructs and transformed them in pairs into yeast strain AH109. The resultant Trp^+^Leu^+^transformants were able to grow on the SD-Trp-Leu-His plate and had beta-galactosidase (LacZ) activities (Fig. 7). These results indicated that FgSsn3 directly interacts with Cid1, suggesting that FgSsn3 and Cid1 may function as a CDK-cyclin pair of the mediator complex in *F. graminearum*.

To test whether FgSsn3 also interacts with other mediator components, we generated the bait construct of FgMed8^46^. In yeast transformants expressing the FgSsn3 prey and FgMed8 bait constructs, growth on SD-Trp-Leu-His plate and beta-galactosidase activities also were observed (Fig. 7), indicating that FgSsn3 may also interact with other components of the mediator complex in *F. graminearum*.

### FgSsn3 positively and negatively regulates different subsets of genes in *F. graminearum*

To identify genes regulated by FgSsn3 in *F. graminearum*, we conducted RNA-seq analysis with RNA samples isolated from 36 h CM cultures of the wild-type strain PH-1 and the *Fgssn3* mutant M9. In total, 2839 genes had over two-fold differences in expression levels between PH-1 and M9, including 1348 and 1491 genes that were up- and down-regulated, respectively, in the *Fgssn3* mutant. Among them, 259 were specifically expressed in *Fgssn3* and 196 were only expressed in PH-1. These results indicate that, similar to yeast Ssn3, FgSsn3 may be functionally related to the mediator complex to repress or activate the transcription of different subsets of genes in *F. graminearum*.

Among the 1348 genes that were up-regulated in *Fgssn3*, Blast2GO (https://www.blast2go.com) analysis showed that genes related to the cellular component ‘membrane, mitochondrion, and mitochondrion envelope’ were enriched. Genes belonging to the molecular function go term ‘transmembrane transporter activity and oxidoreductase activity’, and the cellular process go term ‘monocarboxylic acid metabolic process, generation of precursor metabolites and energy, cofactor metabolic process, and transmembrane transporter also were enriched (Fig. S2).

To our surprise, *TRI13* and *TRI14*, two genes involved in DON biosynthesis were up-regulated over 19-folds in *Fgssn3* mutant (Table S1). It is likely that the regulation of *TRI* genes was de-repressed in CM cultures when *FgSSN3* is deleted. In fact, a number of genes related to secondary metabolism (Table S1), including *PKS10* and *NRPS1*^48^ also had up-regulated expression levels in the *Fgssn3* mutant. These results suggest that *FgSSN3* may be involved in suppressing the expression of genes related to secondary metabolism in vegetative hyphae harvested from 36 h CM cultures.

### Alternative splicing of *FgSSN3*

In RNA-seq data of PH-1, two transcripts of *FgSSN3* derived from alternative splicing of its only intron were detected (Fig. S3). Transcript A had the intron retention and was predicted to encode a protein that is 13-aa shorter than transcript B (Fig. S3A). RT-PCR analysis further verified the presence of transcript A and B in hyphae and perithecia (Fig. S3B). To determine differences in the abundance of two *FgSSN3* transcripts, we examined the expression levels of transcript A and B in RNA-seq data of conidia, hyphae harvested from YEPD medium, and perithecia collected as 8 days post-fertilization. Whereas transcript A was the predominant transcript of *FgSSN3* in conidia, and vegetative hyphae, transcript B was over 2 folds more abundant than transcript A in perithecia (Fig. S3C), suggesting that transcript A mainly functions in vegetative growth and B in sexual reproduction.

## Discussion

In eukaryotic organisms, the mediator complex directly bind to RNA polymerase II to regulate the transcription of various genes^22^. Ssn3 is a nonessential subunit of the mediator complex that is conserved between yeast and humans^46^. Like *SSN3* in yeast, deletion of *FgSSN3* is not lethal but important for hyphal growth and germ tube elongation in *F. graminearum*. In the fission yeast, the generation time of the *ssn3* mutant was longer than that of the wild-type strain^24^. It is possible that the *Fgssn3* mutant also had a longer generation time, which may be related to reduction in growth rate. Interestingly, the growth rate reduction of the *Fgssn3* mutant was nutrient dependent. The reduction in growth was more significant on synthetic media than on media with natural substrates. In yeast, Ssn3 also inhibits yeast filamentous growth in rich medium by phosphorylation of Ste12 and decreasing its stability^23^.

Although it was reduced in growth, the number of conidia produced by the *Fgssn3* mutant was not reduced. In fact, conidiophore development was de-repressed in juvenile CMC cultures. As early as 12 h after inoculation, clusters of conidiophores were observed in the mutant CMC cultures. The up-regulation of *PCS1* and *HIF1* expression^34^,^35^ may be related to conidiophore formation in juvenile cultures. Nevertheless, we noticed that conidia produced by the mutant had abnormal morphology. Unlike normal 5–7 celled conidia produced by the wild type, *Fgssn3* conidia vary from 1 to 4 compartments. We also noticed that many of the conidium compartments contained more than one nucleus. Therefore, *FgSSN3* must also play a role in mitosis and cytokinesis during conidium development.

Although *SSN3* orthologs are well conserved in plant pathogenic fungi, none of them have been shown to be related to pathogenesis. Our data showed that *FgSSN3* is critical for plant infection. The disease index of the *Fgssn3* mutant was less than 1, showing that it was defective in both causing symptoms in the inoculated kernels and spreading via the rachis in infected wheat heads. One contributing factor to its defects in plant infection could be related to the reduction in growth rate. However, the *Fgssn3* mutant also was reduced in hyphopodium formation and penetration of lemma epidermal cells. In addition, the *Fgssn3* mutant was significantly reduced in DON production in diseased wheat kernels and DON is an important virulence factor in *F. graminearum*^4^.

Although *FgSSN3* is dispensable for male fertility, it is essential for female fertility, indicating that it may regulate the expression of sub-sets of genes important for the formation of protoperithecia and other developmental processes related to female fertility. In *F. graminearum*, a number of genes have been reported to be essential for female fertility but dispensable for male fertility, including the *MGV1* and *FgHOG1* MAP kinase and *ZIF1* and *MYT1* transcription factor genes^7^,^14^,^49^,^50^. However, to our knowledge, no mutants are known to be normal in female fertility but defective in male fertility in *F. graminearum*. It is likely that female fertility involving the formation of protoperithecia requires many more genes than male fertility in this homothallic fungus. In the rice blast fungus *Magnaporthe oryzae*, a heterothallic fungus, the *MCM1* transcription factor is essential for male fertility^51^.

No perithecium formation was observed in self-crosses in the *Fgssn3* mutant. In *S. cerevisiae*, *SSN3*, also known as *UME5*, is important for meiosis and sporulation^52^. However, because deletion of *FgSSN3* blocked perithecium formation on mating plates, it is impossible to conclude that *FgSSN3* is important for meiosis and ascospore formation in *F. graminearum*. As an important transcriptional regulator, *FgSSN3* may be involved in the regulation of hyphal fusion and other processes necessary for proto-perithecium development. Because *F. graminearum* is a homothallic fungus, it is also possible that *FgSSN3* is important for switching from vegetative growth to sexual reproduction.

Interestingly, two transcripts of *FgSSN3* were observed in this study with transcript A encoding a 13-aa shorter protein than transcript B due to the retention of an intron in its 5′-UTR. Although the 13 extra amino acid residues at the N-terminal region of FgSsn3B is 39-aa upstream from its kinase domains and unlikely to affect its kinase function, we noticed that transcript A was the predominant transcript of FgSSN3 in conidia, and hyphae but transcript B had a twice more abundant than transcript A in perithecia. Therefore, it remains possible that transcript B plays a stage-specific role in gene expression regulation during sexual reproduction.

In *S. cerevisiae*, the highly conserved aspartic acid residue at position 290 and lysine residue at position 183, are essential for the kinase activity and function of the Srb10 protein^44^,^45^. In *F. graminearum*, the D191A or K71R mutations at the equivalent sites of FgSsn3 produced similar phenotypes as the *FgSSN3* deletion mutant, indicating the importance of kinase activity of FgSsn3 in the function of the protein.

Interestingly, the *Fgssn3* mutant was reduced in DON production but increased in aurofusarin biosynthesis under DON-inducing conditions. Therefore, deletion of *FgSSN3* does not generally blocking secondary metabolism, which is consistent with the fact that *SSN3* has both negative and positive regulatory roles in gene regulation in the budding yeast^46^. *FgSSN3* likely has similar regulatory functions in *F. graminearum*. Our RNA-seq analysis results showed that the transcription of 1348 and 1491 genes was up- and down-regulated, respectively, in the *Fgssn3* mutant. Some genes important for secondary metabolism appeared to be de-repressed in the CM cultures of the mutant, suggesting that *FgSSN3* is involved in the repression of these genes during vegetative growth. To our surprise, two *TRI* genes had increased expression levels in CM cultures, although DON production and *TRI* gene expression were reduced in DON inducing cultures and infected wheat kernels in the *Fgssn3* mutant. These results indicate that regulation of specific subsets of genes by *FgSSN3* may depend on culture conditions, which is consistent with medium-dependent growth defects of *Fgssn3* on different media.

In yeast, Ssn3 interacts with Ssn8 to form a kinase-cyclin pair that functions together with Srb8 and Srb9 as part of the kinase module of the mediator complex^53^. *SSN8* is orthologous to the *CID* gene in *F. graminearum*^47^. Like the *Fgssn3* mutant, the *cid1* mutant was defective in conidium morphology and reduced in growth rate, DON production, and virulence. We further showed that FgSsn3 physically interacted with Cid1. Therefore, FgSsn3 and Cid1 form a similar CDK-cyclin pair that is functionally related to the mediator to affect the transcription by the Pol II holoenzyme in *F. graminearum*.

For transcriptional regulation, Ssn3 can directly phosphorylate the Ser5 of the triple heptapeptide repeats in the CTD of the largest subunit of Pol II^20^. Sequence alignment analysis showed that these phosphorylation sites are conserved in the large subunit of Pol II in *F. graminearum*. Orthologs of many components of the yeast mediator complex also are conserved in *F. graminearum* and other filamentous ascomycetes. Our studies implicate the role of the mediator complex in plant infection, secondary metabolism, and development in a fungal pathogen. In plants, the mediator complex has been shown to be involved in a variety of processes, including defense responses^54^. Considering the importance of the mediator complex in fungal-plant interactions, it will be important to identify and characterize different subsets of genes that are transcriptionally regulated by the FgSsn3-Cid1 CDK-cyclin pair during pathogenesis and the underlying mechanisms related to the mediator complex Pol II activity.

## Materials and Methods

### Strains and culture conditions

The *F. graminearum* wild-type strain PH-1 (NRRL 31084) and all the transformants generated in this study were routinely maintained on PDA plates at 25 °C. Conidiation in liquid CMC medium and growth rate on 5 × YEG, oatmeal agar, and PDA plates were measured as described^47^. Mating on carrot agar plates were assayed as described^9^,^19^. Protoplast preparation and fungal transformation were performed as described^14^. Hygromycin B (Calbiochem, La Jolla, CA, USA) and G418 (Sigma, St. Louis, MO, USA) were added to the final concentration of 300 μg/ml and 400 μg/ml, respectively, for transformant selection. DNA and RNA were extracted from vegetative hyphae harvested from liquid YEPD (1% yeast extract, 2% peptone, 2% glucose) cultures.

### Generation of the *FgSSN3*-GFP, *FgSSN3*^D191A^-GFP, and *FgSSN3*^K71R^-GFP transformants

For generating *Fgssn3/FgSSN3* complemented transformants, a 2.8-kb fragments of the *FgSSN3* gene containing the 1.5-kb promoter region was amplified with primers FGSG_04484/F and FGSG_04484/R and co-transformed with *Xho*I-digested pFL2 vector (carrying geneticin resistance marker) into yeast strain XK1-25 as described^55^. The P_FgSSN3_-*FgSSN3*-GFP fusion construct was identified by PCR and confirmed by sequencing analysis. The D191A and K71R mutations were introduced into *FgSSN3* by overlapping PCR using primers DA/1F and DA/4R, and KR/1F and KR/4R, respectively. The same yeast gap repair approach was used to generate the P_RP27_-*FgSSN3*-GFP, P_FgSSN3_-*FgSSN3*^D191A^-GFP, and P_FgSSN3_-*FgSSN3*^K71R^-GFP constructs. All the GFP fusion constructs were transformed into the protoplasts of the *Fgssn3* mutant M9. The resulting transformants were analyzed by PCR and examined for GFP signals with an Olympus BX-51 epifluorescence microscope (Olympus, Tokyo, Japan).

### Plant infection assays

Conidia harvested from 5-day-old CMC cultures were resuspended to 2.0 × 10^5^ conidia/ml in sterile water for plant infection assay. Flowering wheat head of cultivar Xiaoyang 22 were inoculated with 10 μl conidial suspension at the fifth spikelet from the base of the wheat head as described^55^. Spikelets with typical head blight disease symptoms were examined 14 dpi and disease indexes were calculated^55^. Infection assays with corn stalks and silks of cultivar 2375 were performed as described^56^,^32^. Stalk rot symptoms and discoloration of infected corn silks were examined 14 and 5 dpi, respectively.

### Assays for DON production

The inoculated wheat kernels with typical head blight symptoms were harvested for DON assays as described^56^. DON production in rice cultures^40^ was assayed as described^56^,^57^.

### Assays for penetration and infectious growth

Lemmas were collected from inoculated spikelets at 24 and 48 hpi. After fixation with 4% (vol/vol) glutaraldehyde in 0.1 M phosphate buffer (pH 6.8) overnight at 4 °C, samples were dehydrated in a series of acetone (30, 50, 70, 80, 90, and 100% [vol/vol]). The dehydrated samples were then sputter coated with gold-palladium and examined for penetration structures with a JEOL 6360 scanning electron microscope (Jeol Ltd., Tokyo, Japan). For light microscopy observation, infected lemma and rachis were fixed, dehydrated, and embedded in Spurr resin as described^35^. Thick sections (1 μm) were stained with 0.5% (wt/vol) toluidine blue and examined with an Olympus BX-53 microscope. At least three independent biological replicates were examined for the wild-type and *Fgssn3* mutant strains.

### qRT-PCR analysis

RNA samples were isolated with the TRIzol reagent (Invitrogen, Carlsbad, CA, USA) from conidia, germlings, and perithecia for assaying *FgSSN3* expression, from 12 h YEPD cultures for assaying the expression levels of conidation related genes, and from 6 d DON-inducing cultures containing 5 mM arginine for assaying the expression of *TRI* genes and aurofusarin biosynthesis pathway genes. cDNA was synthesized with the Fermentas First cDNA synthesis kit (Hanover, MD, USA) following the instructions provided by the manufacturer. The beta-tubulin gene *FgTUB2* was used as internal control^56^. Relative expression level of each gene were calculated by the 2^−△△Ct^ method^58^. For each gene, qRT-PCR data from three biological replicates were used to calculate the mean and standard deviation.

### Yeast two-hybrid assays

Protein-protein interactions were assayed with the Matchmaker yeast two-hybrid system (Clontech, Mountain View, CA, USA). ORFs of the *FgSSN3*, *FgMED8*, and *CID1* genes were amplified from the cDNA of PH-1 and cloned into pGADT7 and pGBK7 (Clontech) as the prey and bait constructs. The resulting bait and prey vectors were co-transformed in pairs into yeast strain AH109 (Clontech). The Leu + and Trp + transformants were isolated and assayed for growth on SD-Trp-Leu-His medium and galactosidase activities with filter lift assays^51^. The positive and negative controls were provided in the Matchmaker library construction kit (Clontech).

### RNA-seq analysis

Vegetative hyphae of PH-1 and *Fgssn3* mutant M9 were harvested from 36 h liquid CM cultures. For each strain, two biological replicates were used. Total RNAs were extracted with the Qiagen RNeasy Micro kit and treated with RNase-free DNase I. Complementary DNA libraries with the average insert size of 330 bp were constructed with the Illumina TruSeq RNA Sample Preparation Kit and sequenced with Illumina HiSeq 2000 at the Novogene Bioinformatics Institute (Beijing, China). For each sample, at least 18 Mb paired-end reads were obtained. The resulting RNA-seq reads were mapped onto the reference genome of *F. graminearum* strain PH-1 with Tophat 2.0.12^59^. The number of reads (counts) aligned to each predicted transcript was calculated by FeatureCounts^60^. Differential expression analysis of genes was performed with the edgeRun package^61^ using the UCexactTest function with the Benjamini and Hochberg’s algorithm to control the false discovery rate (FDR). To filter out weakly expressed genes, only genes with a minimum expression level of 1 count per million in at least two samples were included in the analysis. Genes with a FDR of below 0.05 were considered differentially expressed between *Fgssn3* mutant and PH-1. The RNA-Seq data have been deposited in the NCBI Sequence Read Archive database with accession code PRJNA289285.

## Additional Information

**How to cite this article**: Cao, S. *et al*. FgSsn3 kinase, a component of the mediator complex, is important for sexual reproduction and pathogenesis in *Fusarium graminearum*. *Sci. Rep*. **6**, 22333; doi: 10.1038/srep22333 (2016).

## Supplementary Material

Supplementary Information

## Figures and Tables

**Table 1:** Defects of the *Fgssn3* mutant in growth, conidiation, pathogenicity, and DON production.

	Growth rate (mm/d)^a^			Conidiation^b^ (×10^6^ conidia/ml)	Disease Index^c^	DON (ppm)^d^	
	PDA	5 × YEG	OTA			Wheat	Rice
PH-1	12.7 ± 0.1^A^	8.60 ± 0.3^B^	9.9 ± 0.1^A^	1.3 ± 0.3^A^	11.3 ± 1.7^A^	1380.6 ± 80.9^A^	881.7 ± 80.5^A^
M9	9.5 ± 0.1^B^	3.70 ± 0.1^C^	8.6 ± 0.3^B^	1.4 ± 0.1^A^	0.6 ± 0.5^C^	42.6 ± 1.7^B^	2.2 ± 0.2^B^
C1	12.7 ± 0.1^A^	9.58 ± 0.3^A^	10.3 ± 0.3^A^	1.3 ± 0.2^A^	8.3 ± 0.5^A^	1207.7 ± 121.6^A^	844.2 ± 165.2^A^

Data from three replicates were analyzed with the protected Fisher’s Least Significant Difference (LSD) test. The same letter indicated that there was no significant difference. Different letters mark statistically significant difference (P ≤ 0.05).

^a^Average growth rate and standard deviation were calculated from at least three independent measurements.

^b^Conidiation in 5-day-old CMC cultures.

^c^Disease was rated by the number of symptomatic spikeletes 14 dpi. Mean and standard deviation were calculated with results from three independent replicates. At least 10 wheat heads were examined in each replicate.

^d^DON production in infected wheat kernels (Wheat) and rice grain cultures (Rice).
