# Supplementary material for: FgSsn3 kinase, a component of the mediator complex, is important for sexual reproduction and pathogenesis in Fusarium graminearum
Source: Sci Rep. 2016 Mar 2;6:22333. doi: 10.1038/srep22333 (PMC4773989; doi:10.1038/srep22333)
Supplement: Supplementary Information [file srep22333-s1.pdf]

FgSsn3 kinase, a component of the mediator complex, is important for sexual reproduction and pathogenesis in *Fusarium graminearum*

Shulin Cao[1#], Shijie Zhang[1#], Chaofeng Hao[1], Huiquan Liu[1], Jin-Rong Xu[2] and QiaoJun Jin[1]*

[1] State Key Laboratory of Crop Stress Biology for Arid Areas, College of Plant Protection, Northwest A&F University, Yangling, Shaanxi, China.

[2] Dept. of Botany and Plant Pathology, Purdue University, West Lafayette, Indiana, USA.

*Corresponding author:

QiaoJun Jin Northwest A&F University,

Email: jqiaojun@nwsuaf.edu.cn; Tel: 86-29-87081270

[#]These authors contributed equally to this work.

SUPPLEMENTAL FIGURES

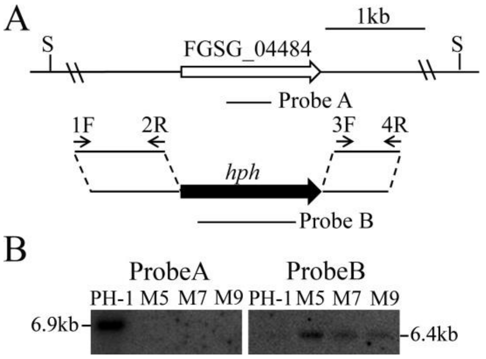

Fig. S1. The *FgSSN3* gene and deletion mutants. **A.** *FgSSN3* locus and gene replacement construct. The *FgSSN3* and hygromycin phosphotransferase (*hph*) genes are marked with empty and black arrows, respectively. S, *Sac*I. **B.** Southern blots of *Sac*I-digested DNA hybridized with fragments of the *FgSSN3* (probe A, left) and *hph* (probe B, right) genes. Lanes left to right: DNA samples from the wild type and transformants M5, M7, and M9.

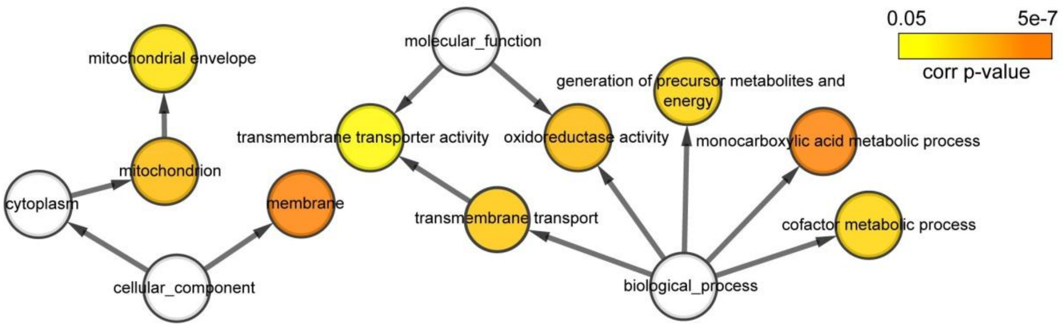

Fig. S2. Go category enrichment analysis of genes up-regulated in the *Fgssn3* mutant.

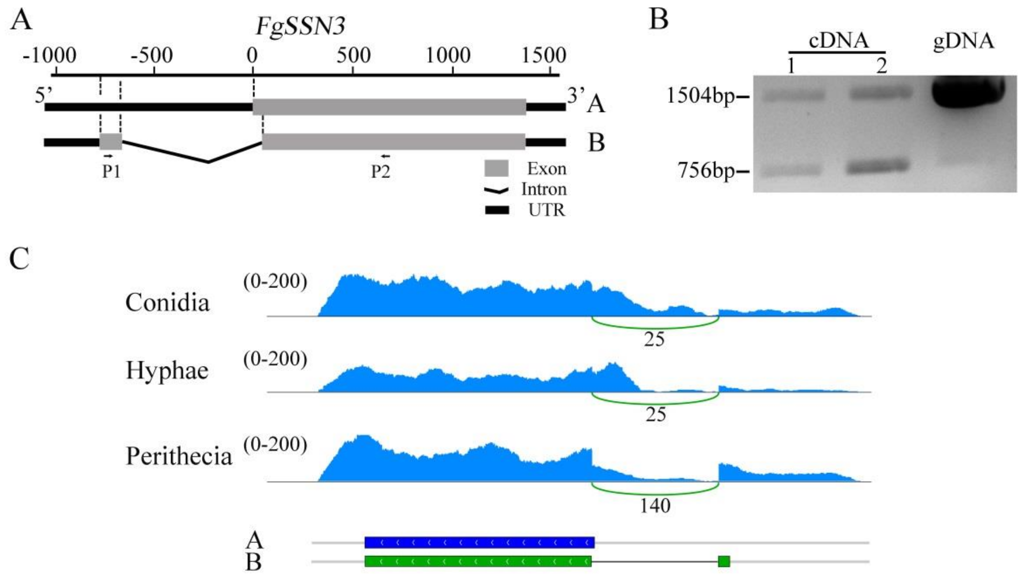

Fig. S3. Alternative transcripts of *FgSSN3*. **A.** Two transcripts derived from alternative splicing of *FgSSN3* were detected by RNA-seq analysis. Transcript A retains a 748-bp intron that is spliced in transcript B. P1/P2, primer pairs used in B. **B.** RT-PCR verification of transcript A and B in hyphae (1) and perithecia (2). **C.** IGV Sashimi plots showing the splice junctions of *FgSSN3* transcripts in RNA-seq alignments of indicated stages. The coverage for each alignment track is plotted as a bar graph. The numbers in brackets indicate coverage threshold. Arcs representing splice junctions connect exons. Arcs display the number of reads split across the junction. The two alternative transcripts are shown below the junction tracks. The RNA-seq data used were available in the NCBI SRA database under accession number SRP062731, which were generated by our lab previously.

SUPPLEMENTAL TABLES

Table S1. Genes related to secondary metabolism that had up-regulated expression levels in the *Fgssn3* mutant

**Gene ID**	**Name**	**Description**
FGSG_03543	*TRI 14*	Tri14 protein
FGSG_03542	*TRI 13*	Cytochrome p450 monooxygenase
FGSG_07798	*PKS 10*	probable polyketide synthase
FGSG_11026	*NRPS 1*	non-ribosomal peptide synthetase
FGSG_02315	*NRPS 4*	related to non-ribosomal peptide synthetase
FGSG_03340	*PKS3*	polyketide synthase
FGSG_08795	*PKS 7*	polyketide synthase
FGSG_02324	*PKS 26*	polyketide synthase that catalyse the condensation of one acetyl-CoA and six malonyl-CoA resultin
FGSG_08209	*NRPS 7*	non-ribosomal peptide synthetase
FGSG_03066	*DTC1*	probable geranylgeranyl-diphosphate geranylgeranyltransferase (AL-2)
FGSG_13153	*NRPS 13*	related to non-ribosomal peptide synthetase
